# Synergistic antimicrobial effect of ascorbic acid and nicotinamide with rifampicin and vancomycin against SCC*mec* type IV methicillin-resistant *Staphylococcus aureus* (MRSA)

**DOI:** 10.1099/acmi.0.000475.v4

**Published:** 2023-02-03

**Authors:** Abdullah AlSaleh, Mohammed Shahid, Eman Farid, Nermin Kamal, Khalid Bindayna

**Affiliations:** ^1^​ Department of Microbiology, Immunology and Infectious Diseases, College of Medicine and Medical Sciences, Arabian Gulf University, Manama, Bahrain; ^2^​ AlSalmaniya Medical Complex, Microbiology Laboratory, Manama, Bahrain

**Keywords:** antioxidant, antibiotic resistance, MRSA, synergy

## Abstract

**Background.** Methicillin-resistant *Staphylococcus aureus* (MRSA) is a pathogenic bacteria involved in a wide spectrum of human diseases. Many virulence factors promote this widespread propagation. One important factor is acquiring antibiotic resistance genes, which leads to a reduction in the availability and efficacy of therapy options. Recently, research has suggested that the remarkable antimicrobial effect of antioxidants against superbugs such as MRSA shows synergistic effects when accompanied by antimicrobial therapy. This paper aims to examine the synergistic effects of ascorbic acid and nicotinamide with a panel of antibiotics used in antimicrobial therapy against MRSA.

**Material and Methods.** Two SCC*mec* type IV MRSA reference strains (EMRSA-15 and USA300) and 10 MRSA clinical isolates feature in this paper. SCC*mec* typing was conducted on the 10 clinical isolates via multiplex PCR after identification. Synergy experiments on antioxidants and antibiotics were evaluated via checkerboard assay. The minimum inhibitory concentration (MIC) of each agent was determined in accordance with the Clinical and Laboratory Standards Institute (CLSI) M100 guidelines through twofold microdilution assay.

**Results and Discussion.** Synergy (FIC <0.5) was demonstrated for ascorbic acid (1/2 to 1/4 MIC) with rifampicin (1/2 to 1/8 MIC), and also ascorbic acid (1/2 to 1/16 MIC) when associated with vancomycin (1/2 MIC). Similarly, nicotinamide (1/2 to 1/16 MIC) showed a synergistic effect when paired with low concentrations of rifampicin (1/2 to 1/16 MIC), and also (at 1/4 to 1/16 MIC) with vancomycin (1/2 MIC). All reduced MICs due to synergistic combinations demonstrated statistical significance (*P*<0.05).

**Conclusion.** The synergistic activity demonstrated in associating antioxidants with antibiotics shows promise in managing superbugs. However, more research is required to better understand the mechanism of the synergy and for utilization in clinical care.

## Data summary

Supplementary materials accompany this paper:

- File S1: extended range of MICs and FICs for all strains and crude statistical analysis.
- File S2: molecular analysis (SCC*mec* typing) of all strains with the reaction conditions used, gel electrophoresis pictures and SCC*mec* type identification key.

## Introduction

Methicillin-resistant *Staphylococcus aureus* (MRSA) is a pathogenic bacterium involved in a wide range of human diseases. The risk goes beyond healthcare settings into the community, and affects individuals without significant risk factors. Many virulence factors promote this widespread propagation, and one important factor is acquiring antibiotic resistance genes, which leads to a reduction in the availability and efficacy of therapy options [1].

Methicillin resistance in *S. aureus* is mainly facilitated via the *mecA* gene that is constituted in the staphylococcal chromosomal cassette (SCC*mec*) genetic element [2]. Furthermore, SCC*mec* elements vary in size amongst MRSA strains and can be categorized into more than 11 types; 1 of the most common is type IV in both healthcare settings (e.g. EMRSA-15) and the community (e.g. USA300) [3].

Recently, antioxidants have been suggested to have a remarkable antimicrobial effect against superbugs like MRSA, showing synergistic effects when accompanied by antimicrobial therapy [4]. This feature is thought to be due to mechanisms such as increasing bacterial cell wall permeability, altering oxido-reduction reactions, decreasing the expression of adhesion molecules and minimizing proinflammatory cytokine production [5–7]. Indeed, investigating supplementary treatment for superbugs to improve the efficacy of current therapies is an important idea. Consequently, ascorbic acid and nicotinamide were considered in this study, not only due to their antimicrobial properties, but also because they are relatively cheap, widely available in a pure form, have low toxicity, and are supported in vegetarian and carnivorous diets, as well as being water-soluble compounds.

This paper aims to examine the synergistic effect of antioxidants (ascorbic acid and nicotinamide) with a panel of antibiotics used in antimicrobial therapy against MRSA.

## Methods

### Bacterial isolates

#### Clinical strains

Ten consecutive, non-duplicate SCC*mec* type IV MRSA strains isolated from skin and soft tissue infections (SSTIs) swabs between December 2020 and April 2021 were obtained from the Microbiology Laboratory at Salmaniya Medical Complex (SMC), Kingdom of Bahrain. All strains were identified as MRSA via the BD Phoenix automated microbiology system (BD Diagnostic Systems, Sparks, MD, USA); SCC*mec* typing was conducted via multiplex PCR in accordance with Boye *et al*. [8]. Primers, reaction conditions and gel pictures can be seen in File S2, available in the online version of this article).

#### Reference strains

In this study, two MRSA reference strains were investigated; EMRSA-15 and USA300 are SCC*mec* type IV MRSA reference strains, obtained courtesy of MRSA Reference Laboratory, Department of Microbiology, School of Medicine, Kuwait University.

### Stock preparation

In this study, two antioxidants (Sigma-Aldrich) were used individually (nicotinamide and ascorbic acid) and eight antibiotics (Sigma-Aldrich) were used (chloramphenicol, ciprofloxacin, linezolid, norfloxacin, oxacillin, rifampicin, tetracycline and vancomycin).

A 1000 µg ml^−1^ stock concentration was prepared for each antibiotic, as mentioned in Andrews [9]. Antioxidants were prepared as needed in stocks of 20 and 500 mg ml^−1^ for ascorbic acid and nicotinamide, respectively. Agents were dissolved according to the conditions listed in Table 1.

**Table 1.** Dissolution conditions for agents used in this study

Agent	Dissolution
Chloramphenicol	Dissolved in absolute ethanol, then diluted with distilled water to maintain a stock concentration of 1000 µg ml^−1^
Ciprofloxacin	Dissolved by glacial acetic acid, then diluted with distilled water to maintain a stock concentration of 1000 µg ml^−1^
Linezolid	Dissolved in distilled water
Norfloxacin	Dissolved by glacial acetic acid, then diluted with distilled water to maintain a stock concentration of 1000 µg ml^−1^
Oxacillin	Dissolved in distilled water
Rifampicin	Dissolved in methanol, then diluted with distilled water to maintain a stock concentration of 1000 µg ml^−1^
Tetracycline	Dissolved in methanol, then diluted with distilled water to maintain a stock concentration of 1000 µg ml^−1^
Vancomycin	Dissolved in distilled water
Nicotinamide	Dissolved in distilled water
Ascorbic acid	Dissolved in distilled water

### Checkerboard assay

The synergistic activity between antibiotics (compound A) and antioxidants (compound B) was tested via checkerboard assay in accordance with Garcia and Isenberg [10]. Each antioxidant (compound B) was paired with one antibiotic (compound A); thus, eight combinations were employed per antioxidant (Table 2).

**Table 2.** Compounds used in the checkerboard assay

Compound A (starting concentration)	Compound B (starting concentration)
Chloramphenicol (128 µg ml^−1^)	Ascorbic acid (10 mg ml^−1^)
Ciprofloxacin (128 µg ml^−1^)	Nicotinamide (250 mg ml^−1^)
Linezolid (16 µg ml^−1^)	
Norfloxacin (128 µg ml^−1^)	
Oxacillin (128 µg ml^−1^)	
Rifampicin (1 µg ml^−1^)	
Tetracycline (4 µg ml^−1^)	
Vancomycin (4 µg ml^−1^)	

**Fig. 1.**
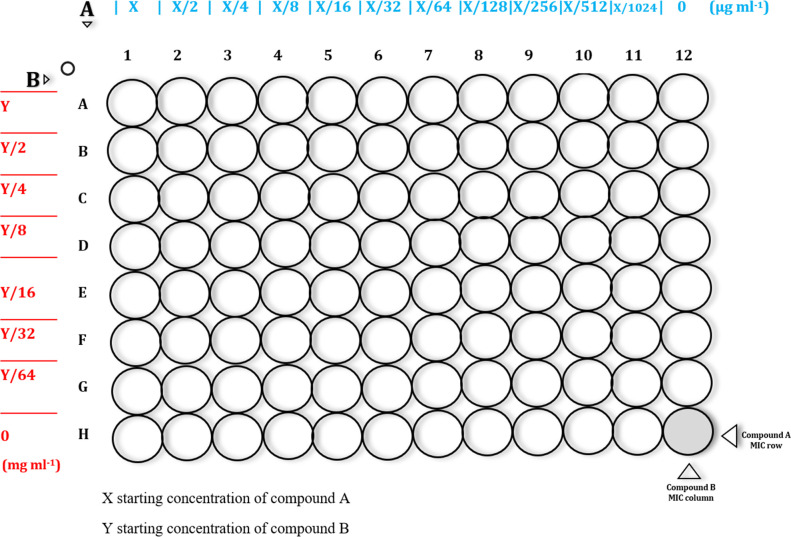
Checkerboard assay template.

A twofold serial microdilution was prepared in Muller–Hinton (MH) broth (Sigma-Aldrich) for each compound and dispensed in the corresponding well in a microtitre plate (Fig. 1). All combinations were tested in triplicate. The lowest concentration preventing turbidity development after 24 h was regarded as the minimum inhibitory concentration (MIC). Microtitre plates were analysed via a Titertek Viewer plate reader (Titertek-Berthold, Germany).

The MICs of compounds A and B were determined in row H and column 12, respectively (Fig. 1). Bacterial inoculum at 1×10^5^ colony-forming units (c.f.u.) ml^−1^ was added to the microtitre plates in accordance with the Clinical and Laboratory Standards Institute (CLSI) guidelines, and the well at 12 h served as a positive control [11].

The microtitre plates were incubated at 37 °C for 24 h to determine the fractional inhibitory concentration (FIC). The ∑FIC was calculated as follows:

$$\Sigma FIC=FIC_{A}+FIC_{B}=\frac{C_{A}}{MIC_{A}}+\frac{C_{B}}{MIC_{B}}$$

where MIC_A_ and MIC_B_ are the MICs of compound A and B alone, respectively; and C_A_ and C_B_ are the concentrations of the compounds in combination, respectively, in the well corresponding to an MIC.

Cutoffs of ∑FIC:

synergy = <0.5

antagonism = >4

additive or indifference=0.5–4

∑FIC_min_=the lowest calculated value of fractional inhibitory concentration

∑FIC_max_=the highest calculated value of fractional inhibitory concentration

### Statistical analysis

Statistical analysis (descriptive statistics and the unpaired *t*-test) was conducted using Microsoft Excel 365. All experiments were performed in triplicate and the data are expressed as the mean±sd whenever applicable. Differences between values were considered to be significant when the *P*-value was <0.05. Statistical analysis data are included in File S1.

## Results and discussion

Ascorbic acid (vitamin C) and nicotinamide (vitamin B3) are essential micronutrients widely used in the food, pharmaceutical and cosmetics industries. Currently, ascorbic acid is one of the most widely used vitamin supplements in the world, and has demonstrated remarkable antimicrobial activity that has been shown to result from many factors, notably its ability to damage bacterial DNA and plasmids [12–14]. Similarly, nicotinamide has shown positive prognosis, especially against *Mycobacterium tuberculosis* infections and dermatological conditions [15, 16].

**Fig. 2.**
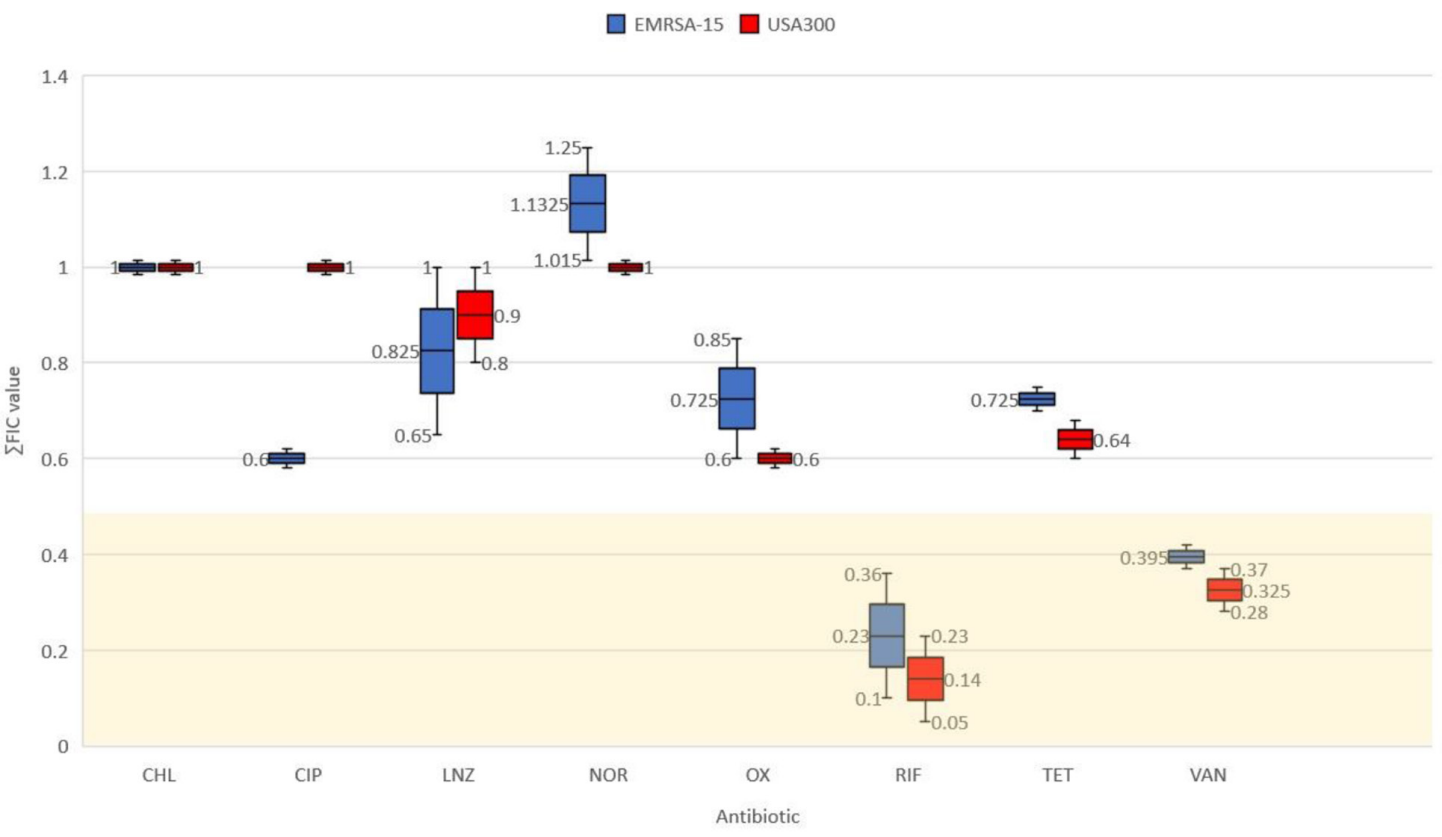
∑FICmin results of incorporating ascorbic acid with antibiotics, synergistic FIC values falling in the gold region <0.5. The mean (& median) is represented by the line inside the box, and the whiskers represent the standard error bars.

**Fig. 3.**
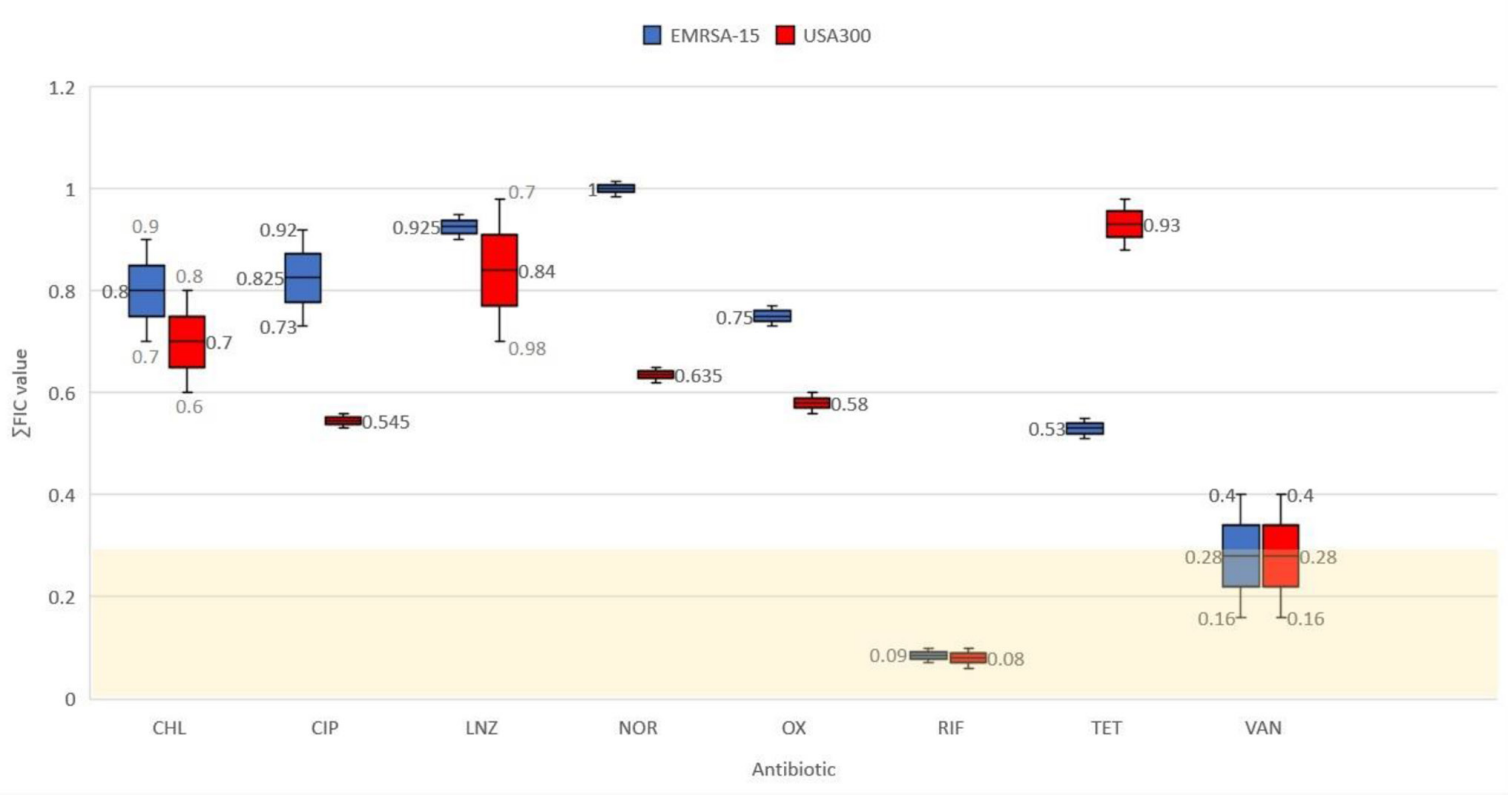
∑FICmin results of incorporating nicotinamide with antibiotics, synergistic FIC values falling in the gold region <0.5. The mean (& median) is represented by the line inside the box, and the whiskers represent the standard error bars.

In the present study, ascorbic acid and nicotinamide were tested individually for synergistic activity with a panel of antibiotics, including chloramphenicol, ciprofloxacin, linezolid, norfloxacin, oxacillin, rifampicin, tetracycline and vancomycin, against SCC*mec* type IV MRSA strains. Synergy (FIC<0.5) was demonstrated for ascorbic acid (Fig. 2) and nicotinamide (Fig. 3) when associated with rifampicin and vancomycin via checkerboard assay. Consequently, demonstrated synergistic combinations were applied to 10 clinical isolates from the same SCC*mec* type for further verification (Table 3). The MIC of each agent (Table 4 and File S1) was determined via twofold serial microdilution in accordance with CLSI guidelines [17].

**Table 3.** ∑FIC_min_ values of the checkerboard assay conducted for 10 clinical isolates

Isolate	Ascorbic acid				Nicotinamide			
	Rifampicin		Vancomycin		Rifampicin		Vancomycin	
	∑FIC_min_	sd	∑FIC_min_	sd	∑FIC_min_	sd	∑FIC_min_	sd
1	0.4	0.1	0.4	0.08	0.1	0.08	0.3	0.05
2	0.2	0.03	0.5	0	0.1	0.04	0.5	0.03
3	0.3	0.1	0.3	0.03	0.1	0.05	0.3	0.03
4	0.4	0.1	0.3	0	0.1	0	0.3	0.18
5	0.4	0.13	0.7	0.13	0.1	0	0.3	0
6	0.4	0.07	0.8	0.2	0.1	0.03	0.3	0.05
7	0.5	0.18	0.4	0.15	0.1	0	0.4	0.1
8	0.3	0	1	0.15	0.1	0.02	0.4	0.06
9	0.3	0.1	0.4	0.5	0.1	0.15	0.4	0.01
10	0.4	0.15	0.3	0.05	0.5	0.2	0.6	0.2

**Table 4.** MIC values for tested MRSA strains

Agent	EMRSA-15	USA300	Clinical isolates	
			MIC	SD
Chloramphenicol (µg ml^−1^)	8	16	–	
Ciprofloxacin (µg ml^−1^)	0.25	16	–	
Linezolid (µg ml^−1^)	4	4	–	
Norfloxacin (µg ml^−1^)	1	32	–	
Oxacillin (µg ml^−1^)	128	48	–	
Rifampin (µg ml^−1^)	0.5	0.32	0.24	0.16
Tetracycline (µg ml^−1^)	0.5	0.5	–	
Vancomycin hydrochloride (µg ml^−1^)	2	2	1.5	0.5
Nicotinamide (mg ml^−1^)	60	60	60	0
Ascorbic acid (mg ml^−1^)	2.5	2.5	2.5	0

In this study, synergy (FIC <0.5) was demonstrated for ascorbic acid (1/2 to 1/4 MIC) with rifampicin (1/2 to 1/8 MIC) against EMRSA-15, USA300 and 80 % of clinical isolates (Fig. 2 and Table 4). Similarly, 1/2 to 1/16 MIC of ascorbic acid, when associated with vancomycin at 1/2 MIC, demonstrated synergy against reference MRSA strains as well as 60 % of incorporated clinical isolates. Similar findings have been reported, as subinhibitory concentrations of ascorbic acid increased the efficacy of rifampicin and isoniazid against *S. aureus* and *M. tuberculosis* [18, 19]. Further, synergy with chloramphenicol, kanamycin, streptomycin and tetracycline was recorded against multi-resistant *Pseudomonas aeruginosa* [20]. In clinical settings, vitamin C vaginal suppositories showed comparable efficacy to metronidazole against bacterial vaginosis (BV); in fact, another paper recorded a better prognosis for BV when co-administering vitamin C and metronidazole [21, 22]. Additionally, co-administration of ascorbic acid with vancomycin has been associated with preserved renal function and reduced vancomycin-associated nephrotoxicity in animal models and critically ill patients [23, 24].

Likewise, nicotinamide (1/2 to 1/16 MIC) showed a synergistic response when paired with low concentrations of rifampicin (1/2 to 1/16 MIC) against 90 % of clinical isolates, as well as reference strains (Fig. 3 and Table 4). In addition, synergy was recorded against 80 % of clinical isolates and the reference strains when associating nicotinamide (1/4 to 1/16 MIC) with vancomycin (1/2 MIC). Unfortunately, we could not find articles in the literature that evaluate the synergistic effect of nicotinamide with antibacterial agents. It is worth noting that the discrepancy in the percentage of clinical isolates affected by the synergistic interaction might be because clinical isolates may harbour various virulence factors that may have hindered the demonstration of synergy.

All in all, there is no concrete explanation as to why ascorbic acid and nicotinamide synergize with the aforementioned antibiotics against MRSA. However, a few theories have been elucidated; antioxidants at subinhibitory concentrations in an iron-rich environment may act as pro-oxidants rather than antioxidants pleiotropically, generating oxidative stress (ROS production) that in turn inhibits bacterial biofilm and capsule formation by inhibiting efflux pumps, and also downregulating biofilm gene expression [25]. In addition, this oxidative stress would impair bacterial cell signalling, lipid alteration and ultimately lead to DNA damage [14, 26]. Even though the aforementioned explanations were examined against *M. tuberculosis *, *Escherichia coli* and *Klebsiella pneumoniae *, a similar paradigm might have occurred in our study, especially because we used MH broth, an iron-rich medium, and further the synergy only manifested at subinhibitory concentrations of the incorporated vitamins (1/2 to 1/16 of MIC). Hence, more research must be conducted to better understand the mechanism behind the demonstrated synergy so it can be used in clinical care.

## Peer review history

### VERSION 3

#### Editor recommendation and comments

https://doi.org/10.1099/acmi.0.000475.v3.1

© 2022 Munnoch J. This is an open access peer review report distributed under the terms of the Creative Commons Attribution License.

**John Munnoch**; University of Strathclyde, SIPBS, UNITED KINGDOM, Glasgow

Date report received: 19 December 2022

Recommendation: Accept

**Comments**: This study would be a valuable contribution to the existing literature. This is a study that would be of interest to the field and community. Dear Abdullah AlSaleh, Thank you for your responses, these are very much appreciated as well as your experimental efforts. Both reviewers and myself are satisfied with your edits and as a result formally accept the manuscript. Best wishes, John.

#### Author response to reviewers to Version 2

**Response to Reviewers:**

Greetings

Thank you considering this manuscript entitled “Synergistic antimicrobial effect of ascorbic acid and nicotinamide with rifampicin and vancomycin against SCC*mec*type IV Methicillin-Resistant *Staphylococcus* *aureus*(MRSA)”. We do apologize for the delay, but as per your request, the practical and statistical analysis needed repeating. Below are the comments received and the corresponding answers, hopefully, they ease all concerns, and if there are any additional remarks, we are happy to address them.

Regards

**Table:** 

Comment	Response
We would ask that the number of replicates be increased to at least three for the checkered board assays	We repeated the entire experiment, this time in triplicates, which thankfully resulted in similar findings. We understand the scientific importance of conducting experiments in triplicate however, the immense amount of plastic waste resulted from the assay is overwhelming, that is in the beginning we only did the experiment in duplicates (P4L21), (P5-L15)
…for example, figure 1, I would expect information on the primers, their specific target, the expected product sizes (bp), DNA ladder information (i.e. supplier etc.) etc.	All the necessary information were added (Supplementary material 2, Figure 1 and 2)
You will also need to update your figures list section as multiple figure 1s now exist etc.	Supplementary figures subheading is added to the list (P9L7)

### VERSION 2

#### Editor recommendation and comments

https://doi.org/10.1099/acmi.0.000475.v2.1

© 2022 Munnoch J. This is an open access peer review report distributed under the terms of the Creative Commons Attribution License.

**John Munnoch**; University of Strathclyde, SIPBS, UNITED KINGDOM, Glasgow

Date report received: 10 November 2022

Recommendation: Major Revision

**Comments**: This study would be a valuable contribution to the existing literature. The reviewers raise concerns regarding the scientific rigour and experimental design of the work. Dear Dr AlSaleh, Thank you for your considered response and efforts to this point. Many of the reviewers concerns have been addressed. However, we initially had concerns about the replication of the study, which in your rebuttal, you indicated each experiment was carried out in duplicate. In any biological setting we would expect experiments to be carried out, minimum, in triplicate as standard to assess reproducibility. As a result we would ask that the number of replicates be increased to at least three for the checkered board assays. Additionally, for figure legends, a general rule of thumb is that there should be enough information to interpret the figure without accessing the main body of text. While not reiterating the full methods section, for example, figure 1, I would expect information on the primers, their specific target, the expected product sizes (bp), DNA ladder information (i.e. supplier etc.) etc.. This is partly to make the information quickly and easily accessible to readers but also to improve the interpretation of the results. While i appreciate this information is in table 1, it does not replace the nature of a figure legend as a standard practice. You will also need to update your figures list section as multiple figure 1s now exist etc. Best wishes, John.

#### Author response to reviewers to Version 1

**Response:**

First and foremost, we would like to thank the editor and the reviewers for their patience and integral comments. We acknowledge that the data presented by us in the previous manuscript were less than perfect. So, we opted to rework all the tables and figures to remedy the concerns presented in the decision email. Below are the comments received and the corresponding answers, hopefully, they ease all concerns, and if there are any additional remarks, we are happy to address them.

Regards,

**Table:** 

Comment	Response
Please upload figures as separate, high resolution, editable files. Acceptable file types are PDF, GIF, TIFF, EPS, JPEG, PNG, SVG, and PPT. Please ensure the legends are in the main manuscript.	Figures were uploaded separately, and a figure list was added in the manuscript (P9L7-10)
What solvents were used to prepare antibiotic stock solutions?	We included a table (Table-1) that include all the solvents used (P5-6)
Could the authors please elaborate on how they established growth inhibition	Lack of visual representation of turbidity was considered to be negative; no subsequent culturing was necessary because we are investigating inhibitory effect not bactericidal effect (P4L19-23)
How many replicates were performed for each assay?	All experiment were done in duplicates (P4L21), (P5-L15)
Any attempt at statistical analysis of the results.	Descriptive statistics as well as unpaired t test were performed (P5L13-17), (Supplementary material 1)
The authors have provided no results of this PCR to confirm the isolates' typing.	We do apologize for overlooking the importance of including the molecular analysis data, hence, now supplementary material 2 is dedicated to rectify this issue
The authors report a range of FIC values both below and above the 0.5 cutoff for synergy.	These ranges were meant to represent the minimum and maximum values of FIC to examine synergy and antagonism simultaneously. Since it was confusing (rightfully so), we opted to only represent the mean minimum value (FICmin) which basically is the most important value of synergy via the checkerboard assay (Table4), (Supplementary material 1)
Figures 2 and 3 clearly illustrate the FIC values the authors intend to present, however are presented without error bars.	Standard error bars and the mean were added to the figures, with their corresponding numerical values
Can the authors present these values as mean MIC/FIC plus standard error or deviation.	All the values were changed from ranges to means ± standard deviation
If the authors measured the turbidity of the wells at the endpoint of their assay, could they provide this data (e.g. in the form of a heatmap)?	An extended synergy tables was added to supplementary material 1 for all strains included in the study. These table illustrate all the concentrations of all the combinations that yielded FICs under 0.5
I would however like to see justification as to why they chose to examine vitamin C and nicotinamide in particular.	We included the reasoning in the introduction. Basically, they are cheap, abundant and most importantly water-soluble, so they are versatile to work with in microbiological assays (e.g., dissolve in broths and conventional bacterial media). (P3L16-21)
Stock concentrations of antioxidants should be stated	We added the stock concentrations of antioxidants (P4L11-13)
There is no reference for the determination of fractional inhibitory concentration	There was at the beginning of the checkerboard assay paragraph. “Garcia LS, Isenberg HD. Clinical Microbiology Procedures Handbook. Third Edition ed. Washington DC: ASM Press; 2010” (P4L16)
Can the authors comment on why there is no MIC data for clinical isolates against several antibiotics in Table 2?	Because the 10 clinical isolates were not tested against antibiotics other than Rif and Van, however, the clinical AST of these isolates was available, and we incorporated the results into supplementary material 1
There are no labels for axes and no explanation of how the data is presented which is very confusing for the reader	We added labels to the axis and all the necessary elements of the figures (Figure2&3)
In general, the order of antibiotics and data presentation should be consistent across all tables to allow ease of comparison for the reader.	This inconsistency is fixed, now all the tables and figures follow the same order of agents
can the authors comment whether they are referencing the fact that FIC ranges used to demonstrate synergy have an upper figure greater than the cut-off for synergy?	These ranges were meant to represent two different values FICmin and FICmax (synergy vs antagonism), we believe presenting the data in this form have made it very confusing, so, we opted to only present FICmin (synergy indicator) and rework all the tables and figures (table4)
Can the authors comment why they used LB instead of MHB in this study?	We actually didn’t use LB broth we only used MHB in this study, it was all a mistake in writing, because we simultaneously were working on inducing staphylococcal toxins via LB broth incubation for another project, so the names were interchanged in the writing process. Very sorry about that (P4L19)

### VERSION 1

#### Editor recommendation and comments

https://doi.org/10.1099/acmi.0.000475.v1.5

© 2022 Munnoch J. This is an open access peer review report distributed under the terms of the Creative Commons Attribution License.

**John Munnoch**; University of Strathclyde, SIPBS, UNITED KINGDOM, Glasgow

Date report received: 15 September 2022

Recommendation: Major Revision

**Comments**: Dear Dr AlSaleh, Following reviews, there are concerns about the scientific rigor of the work. This is a core foundation especially for work published in Access Microbiology. Please address the comments below. Best wishes,

#### Reviewer 2 recommendation and comments

https://doi.org/10.1099/acmi.0.000475.v1.3

© 2022 Anonymous. This is an open access peer review report distributed under the terms of the Creative Commons Attribution License.

**Anonymous.**

Date report received: 14 September 2022

Recommendation: Minor Amendment

**Comments**: 1. Methodological rigour, reproducibility and availability of underlying data Materials and methods: Stock concentrations of antioxidants should be stated There is no mention of how many replicates of the Checkerboard assay were performed. There is no reference for the determination of fractional inhibitory concentration General comments on data in Tables: Can the authors comment on why there is a range for some MICs and for the majority of FICs in Tables 2 and 3? Following on from above, if the cut-off for additive or indifference for FIC is 0.5-4, and the FIC range provided includes numbers greater than 0.5, how can synergy be specifically stated? (e.g. supplementary material 1, bottom row of USA300- range is 0.156-1) Can the authors comment on why there is no MIC data for clinical isolates against several antibiotics in Table 2? If it is because they are sensitive it should be stated as lack of data could also infer they weren't tested. 2. Presentation of results Graph presentation: Greater explanation of the data is needed. There are no labels for axes and no explanation of how the data is presented which is very confusing for the reader. If these graphs represent the data in supplementary material, how do the numbers in the range translate across? There may be a better way to present the data rather than what looks like a bar graph. More information is needed Supplementary data: Presentation in excel sheets is inconsistent and not labelled clearly. Any highlighted cells in the spreadsheet should be clearly labelled with what the highlight means, in addition to an explanation of what the data is. In general, the order of antibiotics and data presentation should be consistent across all tables to allow ease of comparison for the reader. In supplementary materials 1 and 2, MIC and FIC tabs are reversed, and due to an error there are no antibiotics listed apart from ciprofloxacin in supplementary material 1. In supplementary material 2 under the vancomycin/ascorbic heading there are two columns, one containing " >1 " for all isolated- it is not clear what this means. 3. How the style and organization of the paper communicates and represents key findings Presentation of data has made it hard for the reader to interpret key findings. 4. Literature analysis or discussion Results and Discussion comments: The discussion around "The discrepancy in the percentage of clinical isolates affected by the synergistic interaction might be due to human error" in lines 25 and 27, and lines 27 and 28 "It could also be because clinical isolates might harbour various virulence factors which might have hindered the demonstration of synergy" doesn't fit with the rest of the discussion, which very much implies that synergy findings are demonstrated. The overall message of the discussion is that the findings show synergy, but then these sentences don't seem to fit- can the authors comment whether they are referencing the fact that FIC ranges used to demonstrate synergy have an upper figure greater than the cut-off for synergy? This is not clear and a bit confusing. The language used in Lines 27 and 28 is also a bit confusing. Do the authors mean that there may be virulence factors present which protect against the synergistic effects of combined antibiotic and micronutrient treatment? The authors mention using LB in their discussion, and the CLSI paper referenced in the methods describes using Muller-Hinton Broth. Can the authors comment why they used LB instead of MHB in this study? 5. Any other relevant comments It is important to identify alternative/supplemental treatments for AMR infections. From this standpoint the aims of this paper are relevant and timely.

*Please rate the manuscript for methodological rigour*

Poor

*Please rate the quality of the presentation and structure of the manuscript*

Poor

*To what extent are the conclusions supported by the data?*

Partially support

*Do you have any concerns of possible image manipulation, plagiarism or any other unethical practices?*

No

*Is there a potential financial or other conflict of interest between yourself and the author(s)?*

No

*If this manuscript involves human and/or animal work, have the subjects been treated in an ethical manner and the authors complied with the appropriate guidelines?*

Yes

#### Reviewer 1 recommendation and comments

https://doi.org/10.1099/acmi.0.000475.v1.4

© 2022 Mark D. This is an open access peer review report distributed under the terms of the Creative Commons Attribution License.

**David R Mark**; University of Strathclyde, SIPBS, UNITED KINGDOM

https://orcid.org/0000-0002-1688-5224

Date report received: 05 September 2022

Recommendation: Major Revision

**Comments**: 1. Methodological rigour, reproducibility and availability of underlying data (P.5. L.5-L.10) What solvents were used to prepare antibiotic stock solutions? (P.5. L.12 - P.6. L.7) With regards to the checkerboard assay, the preparation of the assay is adequately explained, however could the authors please elaborate on how they established growth inhibition - did they count colony forming units or measure optical density. If they assayed inhibition using a plate reader, what plate reader? There is also no mention of if/how many replicates were performed for each assay, nor any attempt at statistical analysis of the results. 2. Presentation of results Despite carrying out multiplex PCR to type their 10 clinical isolates, the authors have provided no results of this PCR to confirm the isolates' typing. (P.11) Tables 2 and 3 communicate the MIC and FIC values clearly, however I disagree with the authors' argument that vitamin C and nicotinamide are showing synergy with rifampicin and vancomycin (P.8). This is because, in Table 3, the authors report a range of FIC values both below and above the 0.5 cutoff for synergy. (P.12) Figures 2 and 3 clearly illustrate the FIC values the authors intend to present, however are presented without error bars. (Supplementary Tables 1 and 2) Referring back to my comment on the number of replicates performed on the checkerboard assay for each strain, the MIC and FIC values for the compounds tested by the authors are presented as ranges. Can the authors present these values as mean MIC/FIC plus standard error or deviation. If the authors measured the turbidity of the wells at the endpoint of their assay, could they provide this data (e.g. in the form of a heatmap)? This would make the data easier to interpret. 3. How the style and organization of the paper communicates and represents key findings The paper is logically ordered and structured, however the absence of evidence of the authors' typing of their clinical isolates is conspicuous. They clearly explain their logic and the experimental design is sound and clearly explained apart from the concerns I have already raised. 4. Literature analysis or discussion The introduction of the paper is succinct, covering the background of the study and what the authors aimed to achieve by screening antioxidant synergy. I would however like to see justification as to why they chose to examine vitamin C and nicotinamide in particular. The authors discuss nicotinamide's effects on fungal growth, however I am not sure that is a relevant comparison to the work they are presenting here. (P.8.L26-28) The authors postulate that some of their results may have been affected by human error - this is a serious concern and should be addressed to ensure there is no question as to the validity of their results. 5. Any other relevant comments Overall, I believe the work here requires substantial revisions, in particular to show statistical significance of any synergy observed. The authors also need to clarify how they quantified growth in their plates.

*Please rate the manuscript for methodological rigour*

Poor

*Please rate the quality of the presentation and structure of the manuscript*

Satisfactory

*To what extent are the conclusions supported by the data?*

Partially support

*Do you have any concerns of possible image manipulation, plagiarism or any other unethical practices?*

No

*Is there a potential financial or other conflict of interest between yourself and the author(s)?*

No

*If this manuscript involves human and/or animal work, have the subjects been treated in an ethical manner and the authors complied with the appropriate guidelines?*

No: No human or animal work presented

#### SciScore report

https://doi.org/10.1099/acmi.0.000475.v1.1

© 2022 The Authors. This is an open-access article report distributed under the terms of the Creative Commons License.

#### iThenticate report

https://doi.org/10.1099/acmi.0.000475.v1.2

© 2022 The Authors. This is an open-access article report distributed under the terms of the Creative Commons License.

## Supplementary Data

Supplementary material 1

Supplementary material 2
