# Supplementary material for: Synergistic antimicrobial effect of ascorbic acid and nicotinamide with rifampicin and vancomycin against SCCmec type IV methicillin-resistant Staphylococcus aureus (MRSA)
Source: Access Microbiol. 2023 Feb 3;5(2):000475.v4. doi: 10.1099/acmi.0.000475.v4 (PMC9996180; doi:10.1099/acmi.0.000475.v4)
Supplement: Supplementary material 1 [file acmi-5-475.v4-s001.pdf]

Molecular Analysis – SCC*mec* typing results:

Table 1 Primers used for SCCmec typing with their corresponding types

Target	Primer	Sequence (5'→'3)	Product, bp	SCC*mec* Types:				
				I	II	III	IV	V
*ccr*A2-B	F	ATTGCCTTGATAATAGCCYTCT	937		✓		✓	
	R	TAAAGGCATCAATGCACAAACACT						
*ccr*C	F	CGTCTATTACAAGATGTTAAGGATAAT	518			✓		✓
	R	CCTTTATAGACTGGATTATTCAAAATAT						
IS1272	F	GCCACTCATAACATATGGAA	415	✓			✓	
	R	CATCCGAGTGAAACCCAAA						
*mec*A-IS431	F	TATACCAAACCCGACAACTAC	359					✓
	R	CGGCTACAGTGATAACATCC						

Multiplex PCR conditions:

Initial denaturation for 4 min at 94°C, followed by 30 cycles of denaturation (30 s at 94°C), annealing (30 s at 55°C) and extension (60 s at 72°C), and a final extension for 4 min at 72°C. Electrophoresis was performed on a 1.5% agarose gel via ethidium bromide staining, afterwards the gel was visualized under ultraviolet illumination

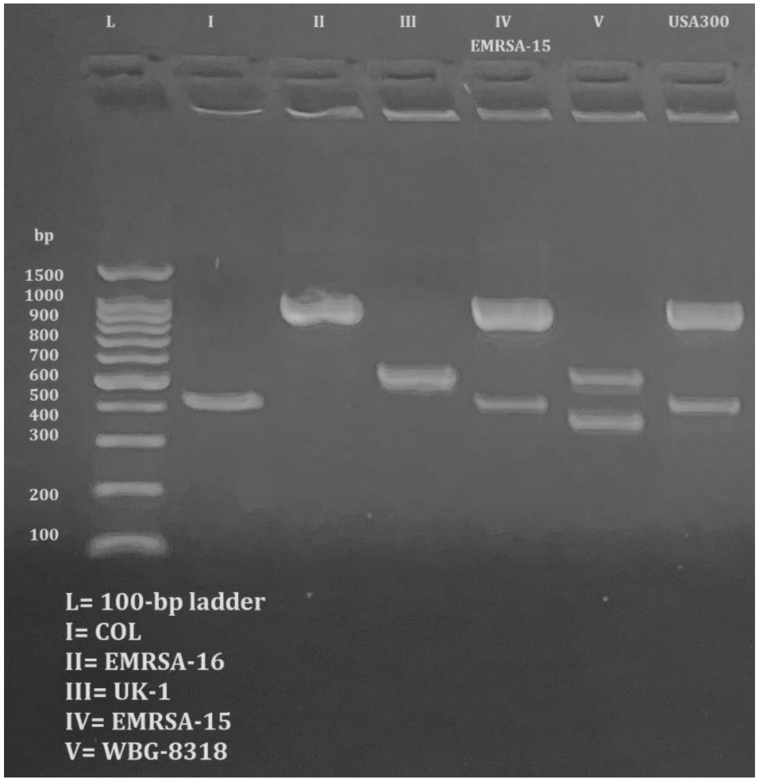

Figure 1 SCCmec typing for the reference strains. Specific genes were targeted in this multiplex PCR assay: IS1272 for type I (415bp), ccrA2-B for type II (937bp), ccrC for type III (518bp), ccrA2-B and IS1272 for type IV (937bp and 415bp, respectively) and ccrC and mecA-IS431 for type V (518bp and 359bp, respectively). 100-bp ladder (Promega, USA) was used in this assay.

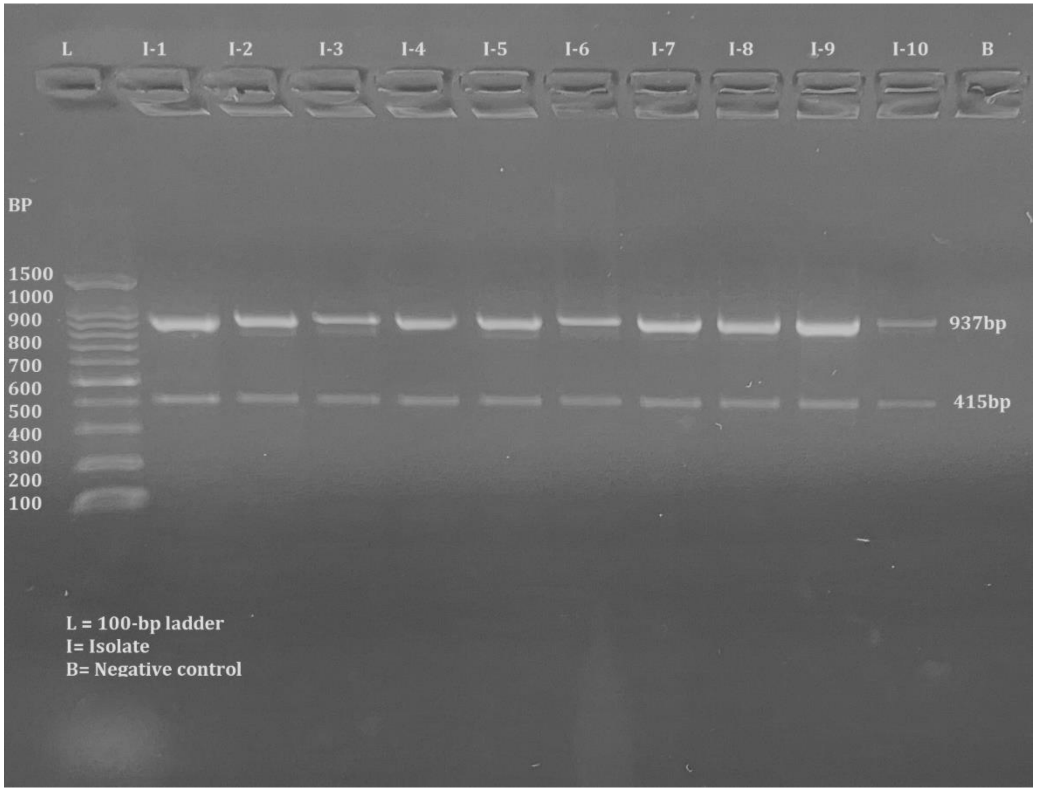

Figure 2 SCCmec typing for the 10 clinical isolates. Specific genes were targeted in this multiplex PCR assay: IS1272 for type I (415bp), ccrA2-B for type II (937bp), ccrC for type III (518bp), ccrA2-B and IS1272 for type IV (937bp and 415bp, respectively) and ccrC and mecA-IS431 for type V (518bp and 359bp, respectively). 100-bp ladder (Promega, USA) was used in this assay.
